# Common and distinct brain functional alterations in pharmacotherapy treatment-naïve female borderline personality disorder patients with and without auditory verbal hallucinations: a pilot study

**DOI:** 10.1007/s00406-020-01102-5

**Published:** 2020-02-03

**Authors:** Chuanjun Zhuo, Feng Ji, Xiao Lin, Hongjun Tian, Lina Wang, Yong Xu, Wenqiang Wang, Baoliang Zhong, Xiaodong Lin

**Affiliations:** 1 School of Mental Health, Jining Medical University, Jining, 272119 Shandong China; 2 Psychiatric-Neuroimaging-Genetics Laboratory, Wenzhou Seventh People’s Hospital, Wenzhou, 325000 Zhejiang China; 3 Psychiatric-Neuroimaging-Genetics-Comorbidity Laboratory, Tianjin Mental Health Centre, Mental Health Teaching Hospital of Tianjin Medical University, Tianjin Anding Hospital, Tianjin, 300222 China; 4 Department of Psychiatry, First Hospital/First Clinical Medical College of Shanxi Medical University, Taiyuan, China; 5 MDT Center for Cognitive Impairment and Sleep Disorders, First Hospital of Shanxi Medical University, Taiyuan, 030001 Shanxi China; 6 Co-Collaboration Laboratory of China and Canada, Xiamen Xianyue Hospital and University of Alberta, Xiamen, 361000 Fujian China; 7 Affiliated Wuhan Mental Health Center, Tongji Medical College of Huazhong University of Science and Technology, Wuhan, 430000 Hubei Province China

**Keywords:** Auditory verbal hallucination, Borderline personality disorder, Global functional connectivity density, Functional connectivity, Wernicke brain

## Abstract

Auditory verbal hallucinations (AVHs) are experienced by approximately 25% of patients with borderline personality disorder (BPD). Despite the high incidence, the pathological features of AVH in BPD remain unclear. This study aimed to investigate whole-brain functional connectivity (FC), as measured by functional connectivity density (FCD), and its relationship with AVH in BPD. 65 pharmacotherapy treatment-naïve female BPD patients (30 with AVH and 35 without AVH), and 35 female healthy controls were investigated. Functional magnetic resonance imaging (fMRI) data were collected to assess whole-brain FC and functional connectivity density mapping (FCDM) was applied to the fMRI data to compute FCD features. Compared to the healthy controls, both BPD groups (BPD–AVH and BPD without AVH) exhibited significantly higher gFCD values in the bilateral prefrontal lobe, bilateral orbital lobule, and bilateral insula, and significantly lower gFCD values in the SMA, right anterior temporal lobule, and the ACC. These altered regions were significantly associated with AVH in the BPD subjects. Moreover, higher gFCD values were observed in the left posterior temporal lobule and posterior frontal lobule. Aberrant alterations also emerged in the left posterior temporal lobule and posterior frontal lobule, mainly in Broca and Wernicke regions. Nevertheless, there was no significant correlation between gFCD values and the severity of AVH as measured by the AVH scores. In summary, we have identified aberrations in the FC and brain metabolism of the aforementioned neural circuits/networks, which may provide new insights into BPD–AVH and facilitate the development of therapeutic approaches for treating AVH in BPD patients.

## Introduction

An auditory verbal hallucination (AVH) is a disturbance in perception, recognized as “hearing voices” or experiencing speech directed at the subjects themselves in an absence of any stimulus from real external language [1]. AVHs are commonly experienced in patients with schizophrenia spectrum disorders and affect approximately 70% of schizophrenia patients. This symptom is also observed in a broad range of other mental illnesses, including major depressive disorder (MDD), bipolar disorder (BD), post-traumatic stress disorder (PTSD) and borderline personality disorder (BPD), as well as in a proportion of healthy individuals [2]. Notably, previous studies have shown that an AVH is more prevalent in BPD, affecting 25–46% of patients, than other common mood disorders such as depression or mania [2, 3]. AVH can elicit the deterioration and reciprocal action with mood disturbance of BPD patients and increase the risk of self-harm and suicidal behaviors.

To date, the pathological features underlying AVH in mental illnesses remain unclear and there is a significant lack of effective treatment strategies. Therefore, it is paramount to better understand the neurobiological models underlying AVH and the specific features of AVH subtypes in different mental disorders. Most recently, Hugdahl and Sommer applied a novel approach termed the levels of explanations (LoE) to better understand AVHs in patients with schizophrenia. This study found that AVHs can manifest at different domains or “levels” of explanation, including the cultural, clinical, cognitive, imaging, cellular, and molecular stages. Neuroimaging data using functional magnetic resonance imaging (fMRI) as well as behavioral observations allow for the exploration of how to identify the treatment target and reduce the intensity of AVHs. Moreover, the neurobiological characteristics obtained from the cellular, molecular, and genetic levels are expected to help in assessing the relationship between changes in neurotransmitters and the severity of AVHs, thereby facilitating the development of new therapeutic agents specific to AVHs. In addition, it has been noted that different types of AVHs have unique clinical features and, therefore, require different treatment strategies. For example, Slotema et al. reported that borderline personality disorder (BPD) patients, experiencing AVHs usually have high suicidal tendencies, are frequently hospitalized, and have high prevalence rates of post-traumatic stress disorder and emotional abuse. Furthermore, the phenomenological aspects are generally similar to those in patients with schizophrenia. Inspired by these important findings, in this pilot study, we explored the common and distinct brain function alterations in BPD patients with and without AVH.

Brain imaging studies have revealed various features of AVH, while focusing primarily on schizophrenia patients. However, although many studies have investigated the brain structural and functional alterations in patients with BPD, these studies also report important findings regarding the pathological features of BPD. For example, some studies found that perturbed activity in the salience network and reward-related circuits also contribute to BPD [4, 5]. Additionally, orbitofrontal overactivation in reward processing was found to be related to various symptoms (suicide, self-injury, and cognitive and emotional distress) of BPD [4-6]. Some studies also reported that the right caudate and left thalamus are key hubs of the abnormal functional network in the BPD patients compared to the healthy controls [7]. Other studies found that the connectivity between the precuneus and frontal regions during rest might be related to extensive processing of internal thoughts and self-referential information in BPD [8], and that the structural and functional abnormality in BPD involves both temporolimbic and frontomedial structures as well as their various connections [9]. Unfortunately, to the best of our knowledge, exploring brain imaging features of BPD–AVH is limited [1, 10].

Although the aforementioned studies support the perturbed network hypothesis in BPD, a majority of these studies have focused on the investigation of the FC strength (FCS) in patients with BPD. Until now, there has been no study to explore the specific number of functional connections (FC number) of AVH in subjects with BPD. Given the high prevalence of AVH in BPD patients and the unidentified pathological mechanisms, there is an urgent need to explore AVH-specific FC aberrant patterns in BPD with the presence of AVH from other perspectives, such as the FC number. In contrast to FCS, FC number is also a pivotal index to assess FC alterations. It has been reported that global FCD (gFCD) can reflect the FC number of one voxel with the other voxels in the whole brain, which can help identify potential disturbances in FC [11]. Simultaneously, gFCD provides an assessment of connection hub(s) in the whole brain without prior knowledge [11-15]. In addition, previous studies have showed that gFCD, in combination with positron emission tomography (PET), can be used to examine alterations in brain metabolism [12, 16-20]. Collectively, gFCD is a useful measure for investigating brain imaging features of DBP-AVH subjects.

In this pilot study, we hypothesized that BPD patients with or without AVH may have common brain connectivity circuit disturbances as well as distinct brain connectivity circuit disturbances. The shared circuit alterations may be related to the common features of BPD, and the distinct alterations may be related to the AVHs experienced by a particular group of patients with BPD. BPD subjects with or without AVH were prospectively enrolled, and fMRI was collected to assess whole-brain FC as measured by gFCD. We attempted to investigate the specific whole-brain FCD alterations and their relationship with AVH in BPD. The results gained through this study may help advance our knowledge about AVH in BPD and assist psychiatrists in making treatment plans.

## Methods

### Human subjects

In the present study, a total of 65 pharmacotherapy treatment-naïve female BPD subjects, and 35 healthy individuals were prospectively recruited between July 2014 and December 2018 at Wenzhou Seventh People’s Hospital. In total, 30 patients, who had endured AVH since BPD diagnosis and which persisted through enrolment in this study, were allocated to the BPD with AVH group, while the remaining 35 who did not experience an AVH episode during the time from the initial diagnosis of BPD to the enrolment in the present study were assigned to the BPD without AVH group. The diagnosis and assessment of BPD were made in accordance with the DSM-IV diagnostic criteria, adopting the Structured Clinical Interview for DSM disorders (SCID) methodology, during which all patients were interviewed by two expert psychiatrists. The healthy control women were screened by two psychiatrists following the Structured Clinical Interview for DSM-IV, non-patient (SCID-NP) version. The following inclusion criteria for the AVH–BPD subjects were used: (1) fully satisfy BPD diagnosis; (2) fully satisfy the AVH diagnosis according to the criteria of Ratcliff et al. [21]; (3) no psychotherapy received within the 3 months prior to enrollment in this study. The exclusion criteria were as follows: (1) neurological diseases; (2) physical diseases that can influence brain functional activity such as serious endocrine system diseases; (3) substance abuse; (4) schizoaffective disorder; (5) major depressive disorder; (6) post-traumatic stress disorder (PTSD); (7) MRI contraindication; (8) claustrophobia; (9) history of unconsciousness for more than 5 min regardless of cause; (10) perimenopausal syndrome (PMS); (11) left-handedness, as assessed with the Annett Hand Preference Questionnaire.

Written informed consent was obtained from all patients and healthy control individuals to participate in this study. This study was reviewed and approved by the Ethical Committee of Wenzhou Seventh People’s Hospital.

### Assessment of mood state and severity of auditory verbal hallucination

The Hamilton Rating Scale for Depression (HAMD) (17-item version) [22] was used to evaluate the mood state of each individual. The auditory hallucination rating scale (AHRS) was applied to assess the severity of AVH symptoms in BPD subjects with AVH, in which the scores of the AHRS (range 0–44) reflect the severity of the AVH(s) and indicate worsening of symptoms [23]. We adopted the Zanarini Rating Scale for Borderline Personality Disorder (ZAN-BPD) to assess the psychopathology of BPD patients [24] and the Montreal Cognitive Assessment (MoCA) [25] to assess their cognitive ability. We further used the Ruminative Response Scale (RRS) to assess the rumination of BPD patients [26].

### Acquisition of magnetic resonance imaging data

MRI data were collected using a 3 T GE Discovery MR750 scanner (General Electric, Milwaukee, WI, USA), which was equipped with an eight-channel phased-array head coil. During MRI scanning, both PBD patients and control individuals were instructed to lie in the supine position without moving his/her head. The following imaging parameters were used: repetition time (TR)—2000 ms; echo time (TE)—45 ms; slices—32; thickness—4 mm; gap = 0.5 mm; field of view (FOV)—220 × 220; acquisition matrix—64 × 64; flip angle (FA)—90°. All MRI data were obtained using parallel imaging with the sensitivity encoding (SENSE) technique (SENSE factor: 2). Structural images were acquired using a high-resolution 3D Turbo-Fast Echo T1WI sequence using the following parameters: TR/TE—8.2/3.2; slices—188; thickness—1 mm; no gap; FA—12°; acquisition matrix—256 × 256; FOV—256 × 256.

### Pre-processing of functional magnetic resonance imaging data

Pre-processing is necessary for fMRI data analysis and statistical evaluation. The statistical parametric mapping 8 (SPM8) software was used to pre-process the data acquired in the resting-state fMRI scans (https://www.fil.ion.ucl.ac.uk/spm). A total of 248 MRI volumes were acquired, of which 238 volumes were analyzed; slice-timing and motion correction was applied after the first 10 functional volumes were discarded to account for scanner stabilization and patient acclimation to the environment. During the pre-processing of the fMRI data, six motion parameters, and the average BOLD signal of the ventricles and white matter, were omitted. Subsequently, the framewise displacement (FD) was measured and data were regressed out of the image stack if the FD of a specific volume was greater than 0.5. The datasets were filtered using a band-pass filter with cutoff frequencies of 0.01 and 0.08 Hz. Individual structural and transformed structural images were co-registered to the mean functional image, and the Montreal Neurological Institute (MNI) space, respectively. With the parameters estimated during the linear co-registration, motion-corrected functional volumes were then normalized to the MNI space. For further analysis, the fMRI images were re-sampled to 3 mm cubic voxels.

### Analysis of global functional connectivity density

The gFCD of each voxel, which is defined as the number of functional connections (FCs) between the given voxel and all other voxels, was calculated using in-house Linux scripts [27-31]. gFCD between voxels were analyzed using the Pearson’s linear correlation assay, in which the correlation coefficient threshold was defined as *R* > 0.6 [27-31]. The gFCD calculations were restricted to the voxels in a cerebral gray matter mask. The gFCD of any given voxel (× 0) was calculated as the number of FCs, denoted as *k*(× 0), between the given voxel (× 0) and other voxels using a “growing” algorithm. The gFCD was subsequently divided by the mean value of the qualified voxels in the whole brain. The gFCD maps were spatially smoothed using a 6 × 6 × 6 mm^3^ Gaussian kernel to minimize variations in the brain functional maps across the study participants. Through comparing the gFCD maps among the three groups, we identified common and distinct gFCD patterns of BPD patients with and without AVH.

### Statistical analysis

Differences in gFCD among the experimental groups were analyzed using a voxel-wise one-way analysis of covariance (ANCOVA), during which demographic factors such as age, gender, and levels of education were included as covariates. This analysis was followed by post hoc intergroup comparisons conducted in a mask that shows gFCD differences from the ANCOVA analysis. Multiple comparisons were corrected for using a family wise error (FWE) approach. *P* < 0.05 was considered statistically significant different. A voxel-wise multiple regression analysis was performed to determine the relationship between gFCD and the AHRS total score of the regions showing significant gFCD differences between the BPD–AVH group and the other two groups. The sociodemographic factors, including age, MoCA, illness duration, HAMD scores, and levels of education were considered nuisance covariates. Given the importance of the AHRS scores in neural correlations, the correlation assay was performed between gFCD and the AHRS scores, and multiple comparisons were corrected for using an FWE method.

## Results

### Demographic and clinical characteristics of the study subjects

The baseline demographics and clinical characteristics of the study subjects, including 30 BPD subjects with AVH, 35 BPD subjects without AVH, and 35 healthy control individuals, are summarized in Table 1. All the BPD subjects, regardless of AVH symptoms, had no impulsive behaviour at the time of assessment and MRI data acquisition. All BPD patients and healthy controls were right handed. The mean duration of illness (DOI) was 140.5 months (SD, 40.3 months) in the BPD with AVH group, and 155.0 months (SD, 51.1 months) in the BPD without AVH group (Table 1). There were no significant differences in age (one-way ANOVA, *F* = 0.210, *P* = 0.833) or education level (one-way ANOVA, *F* = 3.57, *P* = 0.104) among the three groups. No significant differences were found in the severity of depressive symptoms (two-sample *t* test, *t* = 2.859, *P* = 0.149) or DOI (two-sample *t* test, *t* = 1.987, *P* = 0.285) between the BPD–AVH group and BPD without AVH group.

**Table 1:** Demographic and clinical characteristics of the study participants

Characteristics	BPD with AVH group (*n* = 30)	BPD without AVH group (*n* = 35)	Control group (*n* = 35)	*P* value
Age (years): mean (s.d.)	25.5 (3.0)	27.5 (3.5)	27.0 (2.8)	0.833
Duration of illness (months): mean (s.d.)	140.5 (40.3)	155.0 (51.1)	N/A	0.285
Education level (years)	14.0 (2.7)	14.5 (3.2)	14.7 (4.0)	0.104
HAMD scores, mean (s.d.)	3.2 (1.5)	2.9 (1.3)	N/A	0.149
MoCA	25.3 (0.9)	25.6 (0.7)	30 (0.0)	0.011
Auditory Hallucination Rating Scale total score, mean (s.d.)	20.7 (2.9)		N/A	
RRS reflection	13.5 (1.8)	10.0 (2.0)	7.9 (2.7)	< 0.001
RRS brooding	13.0 (2.8)	12.5 (3.1)	8.2 (2.5)	< 0.001
RRS depression	27.5 (3.0)	22.9 (3.8)	18.5 (3.0)	< 0.001

All participants were Han Chinese. A One-way ANOVA was used to test the difference in age across the three groups. A two-sample *t* test was used to compare the differences in duration of illness and HAMD scores between groups

*N/A* not applicable

### Comparative analysis of changes in gFCD across groups

**Fig. 1:**
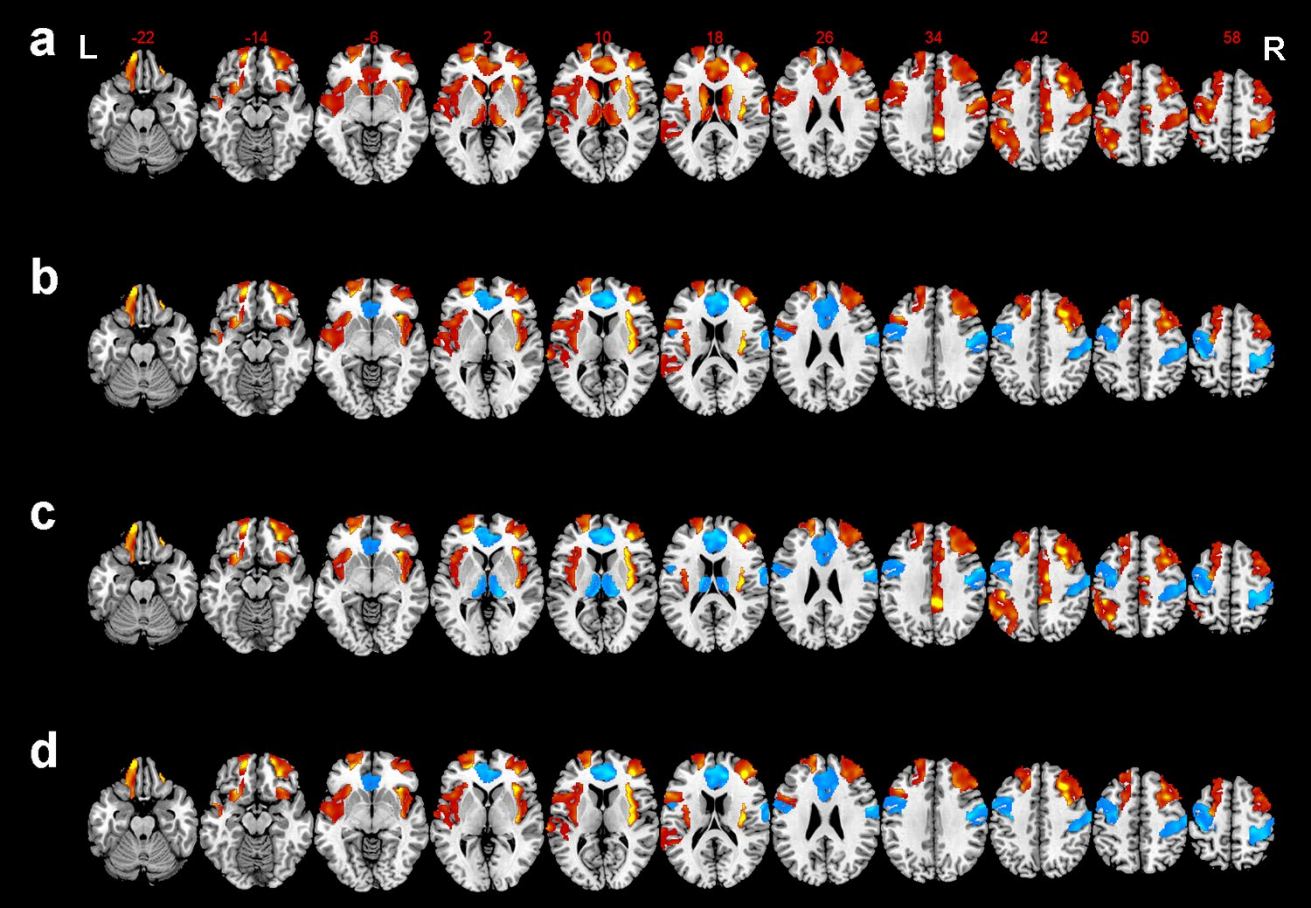
Brain regions illustrating alterations in gFCD across groups. The functional connectivity maps of the three groups, including the BPD with AVH group, the BPD without AVH group, and the healthy control group, were created as described in the “Methods”. **a** Brian regions with significant changes in gFCD among the three groups: BPD with AVH, BPD without AVH, and healthy controls; **b** brain regions with significant changes in gFCD between the two groups: BPD with AVH versus healthy controls; **c** brain regions with significant changes in gFCD between the two groups: BPD without AVH versus healthy controls; **d** brain regions with significant changes in gFCD between the two groups: BPD with AVH versus PBD without AVH. The warm-to-cool color scale in the functional connectivity maps refers to *Z* scores; the warm colors represent an increase in gFCD, while the cool colors represent a decrease in gFCD. The altered regions in the bilateral brain, including the left hemisphere (L) and right hemisphere (R), are illustrated

**Fig. 2:**
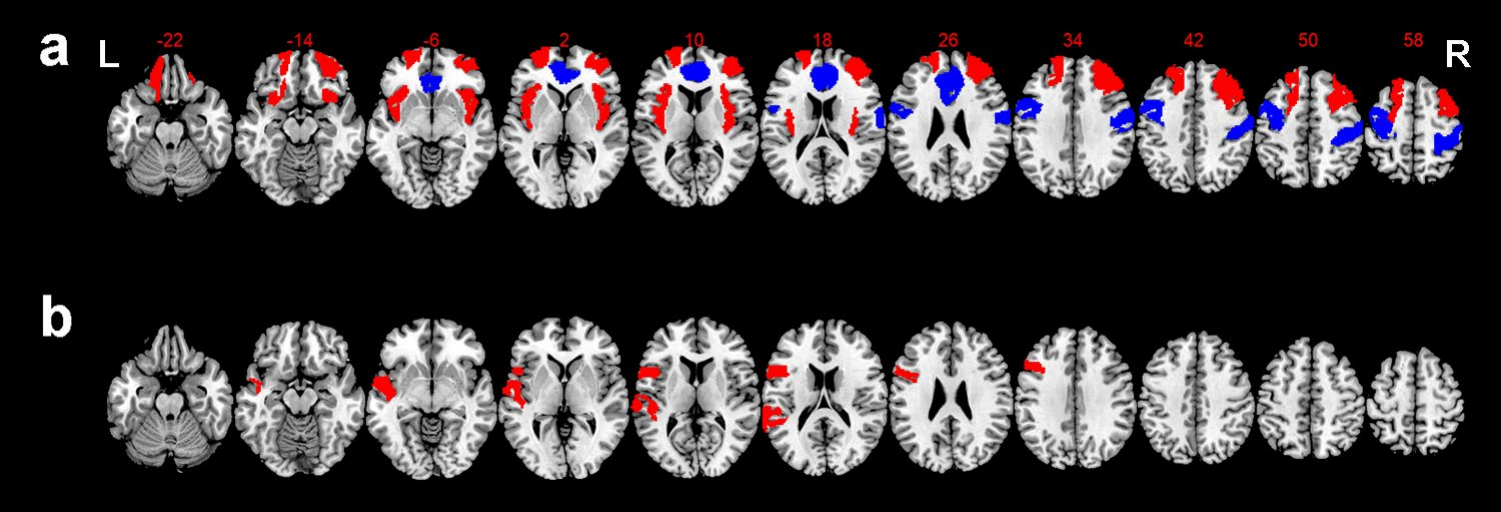
Brain regions illustrating common and distinct alterations in gFCD between two BPD groups. **a** The common gFCD aberrant patterns in the brain regions of the BPD patients with or without AVH. The common gFCD aberrant patterns refer to the brain functional alterations shared by both BPD patients with and without AVH; **b** the distinct gFCD aberrant patterns in the brain regions of the BPD patients with AVH in the BPD–AVH group. The warm-to-cool color scale in the functional connectivity maps refers to *Z* scores; the warm colors represent an increase in gFCD, while the cool colors represent a decrease in gFCD. The altered regions in the bilateral brain, including the left hemisphere (L) and right hemisphere (R), are illustrated

An ANCOVA revealed significant gFCD differences among the three groups in the sensorimotor area (SMA), bilateral prefrontal lobe, bilateral insula, inferior parietal lobule, mid-cingulate cortex, bilateral orbital lobule, bilateral anterior cingulate cortex (ACC), bilateral thalamus, left posterior temporal lobe, left posterior frontal lobe bilateral caudate, and bilateral ventral striatum (FWE corrected, *P *< 0.05) (Fig. 1a). Compared to healthy controls, BPD with AVH subjects showed significantly greater gFCD values in the bilateral prefrontal lobe, bilateral orbital lobule, bilateral insula, left posterior regions of temporal lobule, and left posterior frontal lobule, including Broca and Wernicke regions, but lower gFCD values in the SMA, right temporal pole, and ACC (Fig. 1b). Compared to the healthy controls, the BPD without AVH patients exhibited higher gFCD values in the bilateral prefrontal lobe, bilateral insular, the inferior parietal lobule, the mid-cingulate cortex, bilateral orbital lobule, and lower gFCD values in the regions of SMA, ACC, bilateral anterior temporal lobule, and bilateral thalamus (Fig. 1c). Compared to BPD without AVH, BPD patients with AVH showed greater gFCD values in the bilateral prefrontal lobe, bilateral orbital lobule, bilateral insula, the left posterior regions of temporal lobule, and left posterior frontal lobule, and lower gFCD values in the SMA, right anterior temporal lobule, and ACC (Fig. 1d). Notably, compared to the healthy controls, both BPD groups (BPD–AVH and BDP without AVH) showed greater gFCD values in the bilateral prefrontal lobe, bilateral orbital lobule, and bilateral insular, whereas the gFCD values were significantly lower in the SMA, right anterior temporal lobule, and ACC. As shown in Fig. 2a, these altered regions were significantly associated with AVH in the BPD subjects, and in turn were considered to be the common gFCD aberrant patterns. Moreover, we found a greater gFCD value in the left posterior temporal lobule and posterior frontal lobule, mainly in Broca and Wernicke regions, and we, therefore, defined these regions as the distinct gFCD aberrant patterns of BPD–AVH subjects (Fig. 2b).

### Association between altered gFCD and the severity of AVH in the BPD subjects with AVH

A correlation analysis revealed no significant association of altered gFCD with the severity of AVH, as measured by both the total score and frequency AHRS measures in the DPB-AVH patients. No significant correlation was found between the gFCD and AVH scores, including the total score and single-item scores. Additionally, no significant correlation was found between the gFCD and RRS scores, including the total score and single-item scores, in both BPD groups.

## Discussion

The novel findings obtained in this study of whole-brain FCD, as measured by gFCD in BPD subjects with or without AVH, have important implications for better understanding the neural substrates underlying the psychotic symptoms of AVH in BPD patients. The major results can be summarized as follows: (1) BPD subjects in the two patient subgroups showed significantly altered gFCD compared with healthy individuals; (2) compared to the healthy controls, both BPD groups (BPD–AVH and BDP without AVH) showed greater gFCD values in the bilateral prefrontal lobe, bilateral orbital lobule, and bilateral insular, whereas the gFCD values were significantly lower in the SMA, right anterior temporal lobule, and ACC (Fig. 2a). These alterations were defined as the common brain alterations shared by BPD with and without AVH; (3) compared to the healthy controls and BPD subjects without AVH, BPD patients with AVH demonstrated increased gFCD in Broca and Wernicke regions (Fig. 2b). These alterations were defined as the distinct features of BPD–AVH; (4) no significant association between gFCD and the severity of AVH was observed in the BPD–AVH patients.

Our findings agree with previous studies in the sense that the whole-brain FCD increased in the following regions from a FC number perspective: cingulate cortex, precuneus, prefrontal cortex, insula, superior parietal lobe, thalamus, parietal lobule, and occipital lobe. These alterations in whole-brain FCD are thought to be related to aggression and impulsivity symptoms [5, 32, 33]. Additionally, our new findings, and those of others, converge to implicate hyper-connectivity in the control network, attention network, and reward-processing circuit. The common aberrant patterns in these regions could explain the symptoms shared by BPD patients with or without AVH. Previous studies report that the cingulate cortex, precuneus, prefrontal cortex, insula, superior parietal lobe, thalamus, parietal lobule, and occipital lobe are the key components of the central control network, attention network, and reward processing circuit, and that disturbances in the gFCD in the components of these regions, thereby lead to circuit/network functional disturbance [34-38]. The functional interruption in these circuits/networks have been thought to be associated with some symptoms of BPD, with the control network and attention network functional disturbances related to the aggression and impulsivity symptoms of BPD [34-36]. In addition, the disturbance in the reward-processing circuit has been found to be associated with the emotional regulation disturbance, suicide, and self-harm behavior in BPD [37-47].

We found differences mainly in Broca and Wernicke regions in BPD with AVH versus BPD without AVH and healthy controls and defined these alterations as the distinct gFCD patterns of BPD–AVH. Previous studies have reported that the hyperactivity of these two regions are the basis of the “Top-down effect and bottom-up predictions” hypothesis of AVH [40-42]. It has been proposed that an inappropriate reciprocal action between the bottom-up sensory processing and the top-down regulation processing may interrupt processes governing perception and attention. Bottom-up sensory processing can be defined as perception, and top-down regulation can be defined as attention; the disturbance of this perception and attention network/circuit likely causes AVH [43-45]. Notably, we found that the Wernicke brain region, also commonly known as the language perception region, showed hyperactivity in BPD–AVH patients, which may contribute to their excessive language experience. As the language-generating region, the Broca brain region exhibited hyperactivity, which may have also caused the excessive language experience [46]. As such, there is a possibility that, due to the attention and monitoring deficit (decreased activity in SMA and ACC), the excessive language experience loss ultimately leads to the development of psychotic symptoms of AVH [34, 39, 46]. In summary, our findings of this pilot study are more inclined to support the “imbalance in top-down/bottom-up influences” hypothesis of AVH [35, 41, 43, 45].

It may merit attention in our study that, in the BPD–AVH subjects, the gFCD in the right anterior temporal lobule was highly active, showing hypo-connectivity, whereas in the BPD without AVH subjects, the bilateral temporal lobe was relative inactive with hypo-connectivity. It has been well documented that temporal lobe activity is related to mood state, and that bilateral hypo-connectivity is associated with depressive symptoms [35, 41, 43, 45, 46]. In our study sample, although depressive symptoms did not fulfill the criteria of a depression episode, mood dysregulation was a common symptom of BPD subjects with low mood state and was the main clinical manifestation [47, 48]. Hence, we postulated that the hypo-connectivity of the anterior temporal lobe may be related to depressive symptoms, although no correlation between the gFCD in the anterior temporal lobe and the HAMD score was found in this study, which was likely due to the relatively small sample size. However, compared with the BPD without AVH subjects, the BPD subjects with AVH demonstrated hyperactivity in the left posterior region, for which we postulated this alteration may be associated with AVH in BPD [41, 42, 47]. Moreover, according to the study by Thompson et al., the gFCD also reflects alterations in brain metabolism. Hence, we postulated that the hyper-metabolism in the left posterior temporal lobe and posterior frontal lobe, mainly in the Broca and Wernicke regions represented the pathological features of AVH–BPD subjects [12]. Our findings support the hyperactivity hypothesis of AVH [12].

In this study, we did not find a significant correlation between gFCD and the AVH severity in BPD–AVH patients. This observation was in agreement with previous findings that alterations in brain function were not correlated with the severity of clinical symptoms, for which it has been postulated that these brain alterations could be related to the trait of the symptoms rather than the “state” of symptoms [48-55].

Our study has several potential limitations. First, this study recruited only female BPD patients and female healthy controls. As many previous studies of FC in males and females have shown significant gender differences in various regions of the brain, these differences may affect our whole-brain FCD analyses and their relationship with AVH in BPD. In addition, according to our clinical practice, male BPD patients are less likely to be compliant with fMRI scanning than female BPD patients. With the interesting findings gained in this pilot study of female-only BPD patients, we plan to extend the investigation to male BPD patients. Second, the sample size is relatively small, and this limitation may affect the conclusions drawn from this study. Third, another potential limitation of this study may be related to the evaluation of cognitive ability as previous studies have reported that impairment of cognitive ability was usually observed in BPD patients [40-47]. Fourth, we considered the poor compliance of patients with low cognitive ability in an assessment with MATRICS Consensus Cognitive Battery (MCCB) and, therefore, chose MoCA to evaluate the cognitive ability of subjects in this study, which showed significantly lower scores in BPD patients compared to healthy controls. However, it would be more useful if both MoCA and MCCB were used for the assessment of cognitive ability of patients in future studies, as cognitive ability might influence the common gFCD aberrant pattern. Fifth, although we tried to use the Zanarini Rating Scale for Borderline Personality Disorder (Zan-BPD) to assess the psychopathology of the BPD patients, unfortunately, mainly due to the relatively low education levels of the majority of the participants, we were not able to obtain the qualified assessment and data.

In conclusion, this is the first study, to the best of our knowledge, to examine the gFCD for assessing whole-brain FC in BPD patients with or without AVH. We have identified dysfunctional FC within networks that are significantly associated with the AVH in BPD patients. In addition, the AVH-associated patterns of FC, as measured by gFCD, may offer new insights into BPD–AVH and facilitate the development of new therapeutic approaches for AVH in patients with BPD.
